# Health care encounters in Danish chiropractic practice from a consumer perspectives - a mixed methods investigation

**DOI:** 10.1186/s12998-016-0103-1

**Published:** 2016-07-18

**Authors:** Corrie Myburgh, Eleanor Boyle, Johanne Brinch Larsen, Henrik Wulff Christensen

**Affiliations:** Department of Sports and Clinical Biomechanics, University of Southern Denmark, Campusvej 55, Odense M, 5230 Denmark; Dalla Lana School of Public Health, University of Toronto, Toronto, ON Canada; Nordic Insitute for Chiropractic and Clinical Biomechanics, Odense M, Denmark

**Keywords:** Chiropractic, Consumer behaviour, Health care encounters

## Abstract

**Background:**

Perceived value is the key ingredient to carving and maintaining a competitive business niche. The opportunities to interact with consumers to understand and enhance perceived value are termed ‘touch points’. Due to the out-of-pocket expense incurred by patients, Danish chiropractors are subject to consumer trends and behaviors. The purpose of this investigation was to explore and describe consumer touch points relevant to perceived value through healthcare journeys in chiropractic practices.

**Method:**

We designed a convergent parallel, mixed methods study. Our purposive sampling framework identified 11 chiropractic clinics from which we collected observational field notes, video recordings and face-to-face interviews.

**Results:**

Data was collected between April 14^th^ and June 26^th^ 2014. We described the exteriors and interiors of all participant clinics, interviewed 32 staff members, 12 new patients and 36 follow-up patients and finally video recorded 11 new and 24 follow-up consultations. Categorization and analysis led to the emergence six consumer touch point themes: ‘the internet’, ‘the physical environment’, ‘practice models’, ‘administrative staff’, ‘the consultation sequence and timing’ and ‘a consultation that adds value’. The Internet functions as a tool when choosing/confirming a clinic as appropriate, developing and initial image and managing appointments. The administrative hub appears integral to the shaping of positive consumer experiences outside of the consultation. Clinic location, practice model and interior design may contribute to context effects and thus may influence value perception during the clinical encounter. The duration of hands-on treatment received from the chiropractor is not an apparent consumer focus point. Rather, through a seven stage clinical procedure patients value consultations with clinicians who demonstrate professional competence by effective communication diagnosis/management and facilitating satisfactory treatment outcomes.

**Conclusion:**

At least six consumer touch points add/detract from value-related experiences in chiropractic practices. The duration of hands-on treatment per se does not appear to be a particular focus point. More research is required to explore this issue.

## Background

As in other parts of the world, Danish chiropractors provide conservative health care services to address problems relating to the musculoskeletal (MSK) system [1–3]. Dissimilarly to global norms, Danish chiropractors are mainstream providers with access to public sector practice comparable to that of their physician counterparts [3–5]. Currently; however, only 10% of clinical encounters are likely to occur in publicly funded hospitals and clinics [5]. Thus, societal perceptions of chiropractic practice, as portrayed by patient experiences, develop largely from private practice.

Generally speaking, patients incur an out-of-pocket expense when consulting a Danish chiropractor [6]. Thus, every consultation is an active endorsement of desired fee-for-service. This status quo is interesting from a business and marketing perspective, as the provision of services perceived as valuable to the consumer is the imperative. Therefore, whether they are aware of it or not, it can be argued that chiropractors are subject, to consumer trends and behaviors. However, perhaps due to the historical taboo associated with the financial side of clinical practice, this topic remains under developed within the chiropractic profession [7].

Consumer-centered service provision is a well-described approach to carving and maintaining a competitive value-rich business niche [8]. Integral to such as an approach is a clear understanding of so-called consumer touch points definable as:

All of the communication, human and physical interactions that customers experience during their relationship lifecycle with the healthcare service organization.

Recent, examples of consumer touch points important in health care are web-based consumer/information seeking and consumer satisfaction appraisal [9–11]. In particular, information seeking influences the formation of pre-intervention perceptions and expectations, whereas patient satisfaction appraisal is very useful in building/maintaining high standards of professional practice.

One way of determining how individuals select, access and use chiropractic services is to map typical healthcare encounters. We therefore devised a study as a first attempt to explore and describe consumer touch points relevant to the healthcare journey of chiropractic patients [8].

## Method

### Design

We designed a convergent parallel, mixed methods study, nested within a national census of chiropractors [3], in order to observe healthcare encounters in primary sector Danish chiropractic clinics [12]. In this regard, we simultaneously generated both quantitative and qualitative data from the physical environment (clinic exterior and interior), chiropractors and health care practitioners active in clinics, clinic administrators and patients.

### Sampling

We followed a purposive sampling protocol aimed at striking a balance between the spectrum and commonality of private practice types found in Denmark. We populated a sample matrix using, as criteria healthcare professional mix, national insurance coverage and the chiropractor’s place of education (see Table 1).

**Table 1:** Sample matrix of clinic types accessed using practice model, national health insurance reimbursement and place of education as criteria

Clinic type	Number sampled (*N* = 11)	Mono-professional	Multi-professional	National insurance (Yes)	National insurance (No)	Trained locally	Trained locally and/or abroad
1	1	x		x		x	
2	1	x		x			x
3	1	x			x	x	
4	1	x			x		x
5	1		x	x		x	
6	4		x	x			x
7	1		x		x	x	
8	1		x		x		x

The rationale for these criteria can be summarized as follows: Both mono- and multi-professional practice environments are prevalent in the Danish context [3], potentially resulting in varied patient experiences, especially with respect to patient-centered care [13]. While any licensed chiropractor is entitled to receive reimbursement via the national health care system, the Danish government limits the number of clinics that are registered to do so. As the reimbursement covers 20% of the consultation fee, clinics not operating under the reimbursement system effectively suffer a cost for service penalty and therefore have to devise strategies for attracting and maintaining a patient base. The education received by chiropractors trained locally, differs from those who trained abroad [14]. In particular, the local program is located within the framework of the standard medical education. As such candidates receive a significant part of their formative clinical training in the public sector alongside with physicians. By contrast, chiropractors trained abroad are not typically exposed to other mainstream medical groups during their undergraduate training and learn their patient craft in student clinics, which by and large simulate a private practice-style.

Finally, in order to develop a commonly identifiable consultation sequence, without missing interesting and unusual data from less prevalent clinic types, we oversampled the most prevalent practice type identified through the census, ‘type 6’; that is a multi-professional practice environment that receives national insurance reimbursement and includes chiropractors trained both locally and abroad. [15, 16].

### Data collection

The primary field researcher (JBL), spent one/two days at each of the participating clinics, collecting observational field notes, making video recordings and conducting short face-to-face interviews. Data was collected between April 14^th^ and June 26^th^ 2014.

Fieldnotes were recorded at field sites and were used specifically to describe the physical location and interior design of participant clinics. We included the fieldnotes in the overall data analysis to specifically address research questions.

We used a GoPro Hero 3 digital camera (https://gopro.com/), equipped with a 64GB memory card to continuously record clinical consultations. The camera’s fish eye lens was used to visualize the consultation room. Recording was initiated at the start of practice and continued until data collection seized, thus eliminating the need for the researcher to be present during consultations.

Face-to-face, individual interviews were conducted with patients, clinic administrators, other health care professionals and chiropractors. Patients were interviewed pre- and post consultation, whereas clinic staff were interviewed as they became available. An introduction was provided to assist respondents in focusing on the topic of investigation. A broad, three item interview schedule was constructed around the rationale for seeking care, the services offered in the clinic and whether the internet featured significantly in their experiences. All participants were given the opportunity to respond in Danish; however, after which interviews transcribed in English for analysis.

### Analysis

The data were analyzed by means of substantive categorization, content analysis and thematic analysis. For this purpose, we used computer assisted qualitative data analysis software (ATLAS.ti Version 1.0.21 (91))^(5)^. Once the consultation sequencing had been determined through content analysis, JBL quantified the timing of each consultation and transferred the data in a Microsoft Excel (for Mac -version 14.5.2) spreadsheet for descriptive analysis.

Data were collected confidentially and anonymized for presentation. Furthermore, the study protocol received ethical approval from the scientific ethics committee of the Region of Southern Denmark (jf. § 14, stk. 1-300915) and was conducted in compliance with the stipulations of the Danish Data Protection Agency for the procurement and storage of anonymized interview data.

## Results

Presented in Table 2 below, is a summary of the data collected at each clinic type.

**Table 2:** Summary of data collection by clinic type

Clinic Type (*N* = 11)	Practice description (interior and exterior)	Clinical personnel interviews			Patient Interviews/Video			
		Chiropractor	Other HCP	Secretary	First consult interview	Follow-up consult interview	First consult video	Follow-up consult video
1	1	1		1	1	3	1	3
2	1	2		1	1	2	1	2
3	1	1		1	1	3	1	1
4	1	1		1	1	6	1	4
5	1	2		1	1	3	1	3
6	1	2	2	2	1	4	1	1
6	1	1	1	1	1	3	1	1
6	1	2		1	1	4	1	3
6	1	1		1	1	2	1	2
7	1	1	1	1	1	3	1	1
8	1	2		1	2	2	1	3

Categorization and analysis of our data led to the emergence of 6 themes namely: ‘the internet’, ‘the physical environment’, ‘practice models’, ‘administrative staff’, ‘the consultation sequence and timing’ and ‘a consultation that adds value’.

### The internet

Individuals actively sought out clinics via the Internet, when they were searching for a special interest service provider in their area, such as treatment for colic. Moreover, participants performed a ‘background check’ on clinics through the use of homepage searches and social media websites. Although this behavior was often mainly to satisfy general curiosity, the ability to make and manage appointments online proved, at times, to be a deciding factor for attending the clinic:

[Interviewer]: … why did you visit the home page then?

[Respondent]: It was to see whether it was possible to make bookings online.

It appears that clinic owners who operate a homepage were aware of this tendency and made attempts accordingly to create an inviting image to their clinic:

It’s a fine home page, and I think it reflects nicely what we do without bragging… it means a lot… nowadays that’s the way things are done, computers…

… people find us by way of googling, and everybody who have found us by googling they have visited our home page and looked at it. I also see a lot of new patients who tell me that I could see this or that from your home page.

… it actually turns out that a lot of people have visited it [the website] because they perhaps somehow imagined it [the treatment] to be very violent when a baby is seen by a chiropractor,… it becomes a little demystified, and they are reassured when they watch this treatment video, …

### The physical environment

As is to be expected, clinics were situated in a variety of locations and were either new or well established within the communities they serviced, for example:

The clinic is situated on the first floor in a large building in the center of the town close to the town square and pedestrian areas. A bank occupies the ground floor.

The clinic is part of a bigger health center situated in the old town hall …The health center has one entrance for the medical practices and one entrance for the chiropractic clinic, physiotherapist and podiatrist.

…this is a clinic which has been here for very many years, people have been raised with chiropractic.

This factor influenced the patient mix, which in turn appeared to affect individuals’ clinical expectations and experiences. This becomes more apparent when considering the following two comments by clinic owners:

… I don’t see very many severely chronic patients; I have a lot of acute ones, because they have been seen by their GP, and the GP refers them down here directly,…

and

I actually see a lot of elderly people, but there are many of those in the area around here. …

When probed about the clinic’s decor elements, participants initially tended to trivialize the interior design as an element relevant to their clinical encounter. However, on deeper reflection, our participants reported that a well-appointed interior reflects*’the level of professionalism’* of the clinic and creates a calm atmosphere that *‘make you feel comfortable at being there’.*

Moreover, the clinic interior (and decor) potentially contributed or detracted from soundproofing. In this regard, anonymity was a concern for individuals who noted inadequate soundproofing of consulation rooms. The following comment highlights this issue:

… my only problem with this clinic is that I don’t think you are anonymous because you can hear everything that happens… if I was emotionally affected, if I had been sad or something like that, I would have felt my privacy invaded.

### Practice models

From our observations, it appeared that patients attending mono-professional clinics (chiropractors only) were likely to be exposed to a generalist chiropractic practice model:

… it’s chiropractic for people in all age groups, from babies, we see some who comes here when they are 4 days old, and all the way up to people 90 years old.

A notable exception being special interest services offered for infants:

… I see very many babies because we are two chiropractors in this area… er… which primarily is for the babies from … from, er, the health visitors and directly from the children’s ward at the [name] Hospital. So therefore I probably see more babies than most of my colleagues, but apart from that I think it’s all age groups.

However, in multi-professional practices. patient mix was systematically influenced by the type of national health system reimbursement available. This was particularly the case when physiotherapists were members of the clinical team as physiotherapy is fully reimbursed by the government when patients are treated on referral from hospitals. The following two passages relating to amputee patients highlights this phenomenon:

Two types, I would say. Given we are so many physiotherapists who, er, primarily get their patients referred from the GPs, then it’s, it’s this somewhat older kind of patient that may have had the symptoms for a longer period of time. That’s one type. And the other one is because of the fitness department, due to that we see these young people who have had an injury during exercise. So, that’s the primary ones.

and

The physiotherapists see a lot of cost-free patients … er patients without arms or legs or … we work so close together with the physiotherapists who have many cost-free patients … er, that I perhaps see a group of patients a bit heavier than I did before when I worked in a [different] clinic on the first floor.

### Administrative staff

Patients viewed the clinic’s receptionist as a key element in simultaneously projecting a caring clinical persona and maintaining an efficient patient flow:

… It’s quite cosy here at the clinic. You enter and the receptionist says “Hi [patient name]” right away, and things like that … And there isn’t, there usually isn’t any waiting time, 5 minutes is the most I have seen.

and

It’s important that they are humane down here, and if you don’t show up, I may have forgotten about it one day, well, that wasn’t a problem and I just had an appointment for the following day, and that’s quite different from, more flexible than the public system, right? They will just put you in the back end of the queue, and then you can come back in two months.

Receptionists themselves concur with this view, considering themselves as responsible for *the service* that supports treatment that helps patients *get better*. Part of this service appears to include reinforcing with patients, the clinical competence and caring attitude of clinical staff as well as the strengths of the clinic in servicing their needs optimally. This notion is highlighted by the following receptionist’s responses:

X is very attentive, she is very careful about making her patients feel well and at ease; of course we do what we can, but she isn’t … isn’t superficial, that is that she doesn’t just drag a patient in and pop out again; that is that she never allocates less than 15 minutes for a patient…

and

A broad professional competency… the fact that we have everybody; both physiotherapist, masseur and chiropractor gathered in one place, that it enables good sparring.

### Consultation sequence and timing

From video observation (Table 3), patients spent on average 35 min with their chiropractor during a new consultation and 12.5 min during a follow-up visit. Consultations followed a common sequence of seven activities starting with ‘meet and greet’ and ending in’small talk’.

**Table 3:** Video observation of new and follow-up consultation activities by duration

	First consult	Follow-up consult
	Mean (SD) (Min- Max)	Mean (SD) (Min- Max)
Total duration	34:46 (8:17) (25:47-50:38)	12:24 (03:51) (06:12-18:34)
Meet & greet	00:21 (00:17) (00:00-00:57)	00:14 (00:15) (00:00-00:55)
Patient history	10:38 (06:32) (01:08-25:06)	01:37 (01:22) (01:26-06:27)
Patient evaluation	09:20 (02:56) (03:31-12:21)	02:27 (01:41) (00:28-06:51)
Treatment	06:40 (03:00) (02:30-12:24)	05:03 (03:31) (00:12-15:24)
Advice	06:54 (03:53) (03:33-15:40)	01:45 (02:07) (00:00-08:52)
Exercise prescription	00:24 (00:30) (00:00-01:30)	01:37 (01:55) (00:00-06:59)
Small talk	00:37 (00:58) (00:00-03:11)	01:54 (01:40) (00:08-05:00)

Duration presented in minutes and seconds

**Fig. 1:**
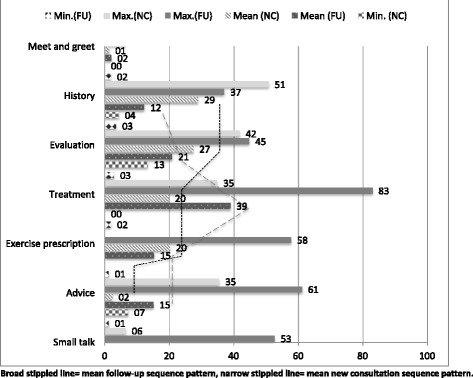
Proportional breakdown of first and follow-up consultations by time

Generally speaking, new consultations revolved around patient history (29%) and evaluation (27%), with a gradual step-wise decrease in proportional amount of time spent on the remaining consultation activities (Fig. 1). In contrast, follow-up visits built up to a peak around treatment (39%). More time was devoted to advice in follow-up visits (15%), compared to the initial consultation (2%).

Notwithstanding the differences in proportions across the various categories (Fig. 1), the actual treatment time varied by less than two minutes between the initial and follow-up consultations (see also Table 3). Clinicians had on average between 5 and 7 min of hands-on treatment.

### A consultation that adds value

In general, our patient participants valued consultations with a practitioner whom they perceived to be professionally competent, who effectively communicated his/her diagnosis and management plan and was able to facilitate a successful treatment outcome. This triad of factors is observable from the following response:

You could feel from the beginning that she knew what she was doing, … I told her that the one I saw and then she got in contact with him, … confidence is established, and then I believe she … and I can feel that it helps.

Successful treatment outcomes were reported as routine improvements associated with ongoing care for example:

I always expect recovery or some kind of relief of the pain and I can feel it in my body afterwards [from] the treatment they give me, …

But also as seminal responses to treatment, such as:

… it improved fantastically already after the first time [treatment] … there was no doubt that I could feel she knew exactly what she was doing.

## Discussion

Our findings confirm the Internet to be a pervasive component of the patient’s health care journey [9, 11]. In this instance, individuals seek out and confirm the appropriateness of a particular clinic through internet searches, develop a set of clinical intervention expectations by visiting websites and enjoy the convenience of online booking systems to plan their consultation schedules. Due to the easy access and abundance of websites posting clinical interventions provided in Danish chiropractic clinics, health care encounters potentially precede arrival at the clinic. Individuals searching for a particular intervention, for example treatment for their colicky child, are particularly likely to be affected in this regard.

Clinics integrated in medical centers/hospitals, those offering special interest services and team-oriented approaches potentially offer niche experiences to patients and perhaps stand in contrast to freestanding, mono-disciplinary settings following a more traditional chiropractic practice model. While 86% of chiropractic clinics in Denmark benefit from government co-payment [3, 5], the level of co-payment averages at around 20% of each consultation. Thus, gaining a competitive edge through team-oriented or special interest practice represents an attractive business strategy [8, 17]. Moreover, clinics not benefitting from government co-payment and who are forced to offset their co-payment disadvantage may be more aggressive in this regard. Further investigation to illuminate this issue is required as inter-clinic competition may be a source of future tension.

Chiropractic patients are exposed to a variety of physical environments, depending on the clinic location and practice model employed. While we found no indication that these factors impacted directly on patient health care experiences, participants offered value-related insights with respect to interior design and soundproofing. This observation aligns with the notion of context effects attributable to the health care setting [18]. Investigations conducted in the aviation industry [19, 20] indicate that passenger comfort is enhanced by feelings of ‘peace of mind’ and ‘wellbeing’. And by reducing cabin ambient noise and making use of innovative use of colours and materials boosts passenger comfort. One might argue then, that a well-planned physical environment similarly, through context effect, ads value to patient health care experiences.

As the primary point of contact clinic reception staff fulfil a vital guiding role to patients on their health care journey. It is interesting to note though that 25% of Danish chiropractic practices do not employ dedicated reception staff [5]. A question that springs to mind then is, whether these chiropractors employ more sophisticated bedside manners in order to facilitate patient-centered care both in an out of the consultation room [21]?

Our observation of consultation times correspond closely to Nielsen et al., who reported new consultation and follow-up consultation durations of 40 min and 13 min, respectively [5].

While proportionally varied, hands-on treatment accounted for a small proportion of the consultations. This stands in contrast with other forms of manual therapy, such as massage, which typically require upwards of twenty minutes to perform [22]. This observation suggests that patient value-related experiences and expectations may not center on the time the chiropractor allocates to hands-on treatment. Rather individuals are more likely to make value judgements regarding professional competence, effective communication and management outcome.

### Strengths and limitations

To the best of our knowledge, this investigation is the first to explore patient health care journey using a mixed methods approach. As a consequence, we were able to describe, but also start providing insights into factors that add value to patients’ health care experiences. This investigation was nested in a national survey and we were therefore able to fill a comprehensive sampling strategy to capture a range of clinical settings.

Our study made use of short pre-post consultation interviews. This placed a limitation on the depth of probing and data saturation. Practitioners may have changed practise style while being video observed. However, despite being blinded to one another practitioners exhibited remarkably similar practice styles. Furthermore, given the range of consultation times and proportional differences we observed, it is unlikely that this resulted in an artificial, systematic modification of behaviour.

## Conclusions

From a value perspective, at least six consumer-related touch points add/detract from value-related experiences in chiropractic practices. The Internet features in three key areas regarding the clinic, namely: choosing/confirming the clinic as appropriate, developing and initial image, and managing appointments. The administrative hub plays an integral role in shaping a positive consumer experiences outside the consultation. Clinic location, practice model and interior design may contribute to context effects thus influencing the perceived value of the clinical encounter. The duration of hands-on treatment received from the chiropractor is not a point of focus. Rather, through a seven stage clinical procedure, patients’ value receiving care from a clinician they perceive as competent and who matches their expectations during the clinical encounter.
